# Persistent Vitello-Intestinal Duct Masquerading as Acute Appendicitis: A Case Report

**DOI:** 10.31729/jnma.8239

**Published:** 2023-08-31

**Authors:** Subita Neupane, Ashutosh Kashyap, Biraj Pokhrel, Roshan Pathak, Dinesh Prasad Koirala

**Affiliations:** 1 Department of Family Medicine and Emergency Medicine, Civil Service Hospital, New Baneshwor, Kathmandu, Nepal; 2 Department of Pediatric Surgery, Tribhuvan University Teaching Hospital, Maharajgunj, Kathmandu, Nepal

**Keywords:** *appendicitis*, *case reports*, *intestinal obstruction*, *laparotomy*, *omphalomesenteric duct*

## Abstract

The vitello-intestinal duct normally regresses with the development. But, in certain cases, it could persist and present as Meckel's diverticulum. Here we report a case of an eight-year-old boy presenting with peri-umbilical abdominal pain, vomiting and loose stool. He was initially diagnosed and managed as acute appendicitis but did not improve, rather developed features of intestinal obstruction. Exploratory laparotomy was done which revealed persistent vitello-intestinal duct and was managed surgically. This case report highlights that in any case of suspected acute appendicitis, the complications of persistent vitello-intestinal duct should be considered as one of the differentials.

## INTRODUCTION

The midgut of early embryo communicates with its yolk sac, as the placental nutrition is yet to be adequately established. This communication is supposed to be obliterated in between fifth to the seventh week of gestation;^1^ however, the duct could persist, either in part or as a whole, and present later as Meckel's diverticulum, vitelline cyst, patent vitelline duct, fibrous band, sinus tract etc.^2^ The persistent duct could cause various problems such as bowel obstruction, gastrointestinal bleeding, umbilical sinus, fistula or hernia and also may mimic acute appendicitis.^3^

## CASE REPORT

In this case, an eight-year-old boy presented to our pediatric emergency with complaints of abdominal pain and multiple episodes of vomiting and passage of loose stools for one day. The pain was periumbilical and spasmodic in nature at presentation. The pain was sudden in onset, non-radiating, aggravated on attempting to eat anything, was not relieved by non-steroidal anti-inflammatory drugs, and was severe enough to bring the boy crying and wriggling in pain. The pain gradually migrated to the right iliac fossa. Along with severe abdominal pain, he also had multiple episodes of vomiting, which was non-projectile and contained food particles, bilious, but not mixed with blood. He also suffered from multiple episodes of watery loose stool, not mixed with blood, for the same duration and pain was not relieved with passage of stool.

On examination, the boy was ill-looking, well oriented to time, place and person. The general examination of the boy was normal. His heart rate was 88 beats per minute, blood pressure was 110/80 mm of Hg, respiratory rate was 22 breaths per minute, temperature was 36.1°Celsius. On abdominal examination, guarding, right iliac fossa tenderness, and rebound tenderness was present.

Baseline investigations were sent and his initial management was done in the emergency. His total leukocyte count was found to be 19,200/mm^3^; and ultrasonography (USG) was not suggestive of anything except mild probe tenderness in right iliac fossa. Abdominal X-ray was not laden with any findings either during initial set of investigations. His 'MANTRELS' (migratory right iliac fossa pain, anorexia, nausea and vomiting, right iliac fossa tenderness, rebound tenderness, elevated temperature, leucocytosis and shift to left) was calculated to be eight. With this clinical presentation in the emergency, the boy was rushed for an emergency open appendectomy.

**Figure 1:**
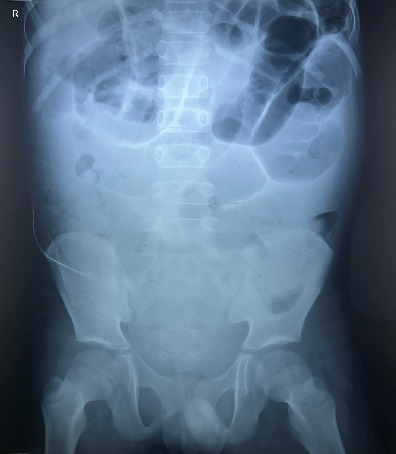
Supine view abdominal X-ray, showing intestinal obstruction.

Intraoperatively, the tip of the appendix, which was retrocecal in this patient, was found to be inflamed; but the base was healthy. There was around 20 ml of serous peri-appendiceal collection. Appendectomy was done but the patient did not get better after the procedure. Instead, his condition started worsening. Meanwhile, conservative management was being done but showed no signs of improvement. He had not passed either stool or flatus even till the second postoperative day. Further, his pulse rate was climbing over 110 per minute, blood pressure was 110/80 mm of Hg, respiratory rate was 25 breaths per minute, and his temperature was 37.1° Celsius. This is when an abdominal X-ray (supine and erect views) was done which revealed signs of intestinal obstruction (Figure 1).

He was rushed in for emergency exploratory laparotomy as his condition worsened clinically.

**Figure 2:**
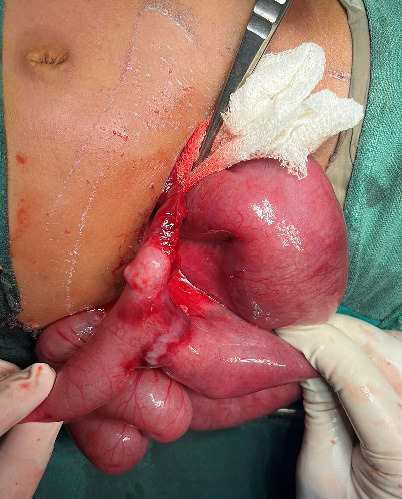
Closed loop obstruction of a segment of ileum due to persistent vitello-intestinal duct.

Incision was given through the same incision made for appendectomy. The skin at the site of incision was healthy. The incision was extended. The appendectomy stump appeared healthy. Further exploration revealed a closed loop obstruction of a segment of ileum (Figure 2).

**Figure 3:**
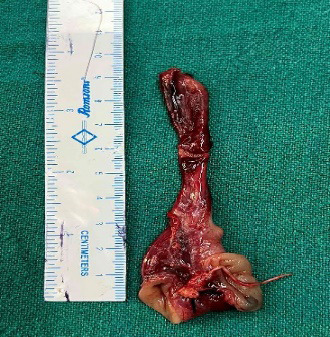
Resected the vitello-intestinal duct along with the adjoining ileal segment.

The obstruction had taken place due to a patent vitello-intestinal duct that was attached to the abdominal wall and the loop of ileum took a twist around this persistent anomaly. Resection of the vitello-intestinal duct along with the adjoining ileal segment (wedge resection), 20 cm proximal to the ileocecal junction was done (Figure 3).

**Figure 4:**
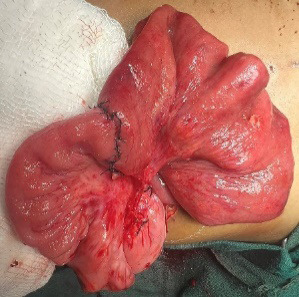
An end-to-end ileal anastomosis.

An end-to-end ileal anastomosis was fashioned and the patency of anastomosis was ensured (Figure 4).

A 16F drain was kept, thorough lavage was done, and the abdomen was closed in layers in half an hour.

The postoperative period was uneventful and he recovered quite well from two surgeries without any postoperative complications. Upon follow-up after two months, he had recovered without any new issues.

## DISCUSSION

One of the presentations of the persistent vitello-intestinal duct is bowel obstruction.^4^ Bowel obstruction manifests itself with classic features of abdominal pain, nausea, vomiting, obstipation and distension, with variations in its presentation depending on the site of obstruction. Among the multitude of patients presenting every day to different emergencies across the globe, closed loop obstructions forms a small minority of cases and is a result of internal hernias, congenital bands, postoperative adhesions and malrotation. A closed loop obstruction is a type of intestinal obstruction in which the bowel is nipped off both distally and proximally, such that a segment of either the small or the large bowel is closed from both ends leading to the obstruction. It should be treated as a of surgical emergency.^5^

Our patient had developed a closed loop obstruction of a segment of ileum secondary to its twisting around the persistent vitello-intestinal duct, leading to dilated bowel loops proximal to the obstruction. The interesting thing about this case is that despite being a bowel obstruction, it initially presented with clinical manifestations pointing towards acute appendicitis.

The pain in acute appendicitis, in its classic form would begin in the periumbilical area to later become localized to the right lower quadrant.^6^ When a patient presents with these symptoms, and the demographic is also compelling, it becomes a knee-jerk reaction to think of acute appendicitis, and this bias can lead to blunders, which occurred in the case. And intraoperatively, as we saw the inflamed tip of appendix and peri-appendiceal fluid we went along with the surgery, never suspecting that it could be something else as well, and looked on for other causes only after the patient did not improve with given management.

Early and prompt diagnosis of this condition is essential as the risk of bowel ischemia is particularly high in closed loop obstructions wherein the bowel is being pinched both proximally and distally.^7^ However, the diagnosis is not always straightforward, especially in case of a virgin abdomen (no abdominal surgery in the past). There can be diagnostic difficulties based on the clinical presentation alone, which happened in our case where we suspected acute appendicitis and operated for the same. Further, plain x-ray of abdomen and USG also do not confer specific signs for small bowel obstruction, especially early on, which happened in our case too.^5^

The management of bowel obstruction is aimed at correction of physiological derangements, proper bowel rest and decompression, and eliminating the source of obstruction. Non-operative management should be tried in case of adhesive bowel obstruction without the signs of peritonitis, strangulation or hernia.^8^ Complete or high-grade obstructions, closed loop obstructions, call for urgent or emergent surgical interventions as the risk of ischemia increases. There is high morbidity and mortality if the diagnosis is delayed.^9^ Hence, emergency laparotomy should be done promptly. Also, some centers mandate laparotomy in cases of virgin abdomen.^10^ Treatment is guided by the patient's clinical condition, and in our case, the boy had started worsening despite us misinterpreting that we had treated the patient, and he was rushed in for exploratory laparotomy wherein we found the obstruction due to the vitello-intestinal duct, and it was managed via wedge resection of duct and end to end ileal anastomosis.

We should always keep our mind and eyes open, despite the presentation and management being seemingly obvious as it is not always what it appears to be in the first glance. A persistent vitello-intestinal duct should be kept as a differential in patient's presenting with closed loop obstruction. The management of these cases should be prompt with wedge resection and anastomosis.
